# Burn patients’ perceptions of skin grafting in China: a single-center retrospective cohort study with paired pre-post assessment

**DOI:** 10.3389/fpubh.2026.1754982

**Published:** 2026-01-23

**Authors:** Pei Xu, Hong Kong, Jiliang Li, Guoying Jin, Shengyong Cui, Sida Xu, Yaohua Yu, Xin Le, Youfen Fan

**Affiliations:** 1 Burn Department, Ningbo No. 2 Hospital, Ningbo, Zhejiang, China; 2 Ningbo College of Health Sciences, Ningbo, Zhejiang, China

**Keywords:** anxiety, burn, fear, nursing, operation education, psychological support, skin grafting

## Abstract

**Objectives:**

This study aimed to quantify changes in psychological perceptions before and after skin grafting and to identify key modifiable factors influencing these perceptions in Chinese burn patients.

**Methods:**

A single-center retrospective cohort study analyzed paired pre- and post-operative survey data from adult inpatients who underwent skin grafting between January 2018 and December 2021. Preoperative (1–3 days before surgery) and postoperative (at discharge) data were extracted from the medical records of 475 patients. Surveys assessed anxiety (Numerical Rating Scale, NRS; Hospital Anxiety and Depression Scale, HADS), fear (NRS), depression (HADS), and perceptions of grafting. Multivariate linear regression controlled for confounders including age, sex, burn site, number of surgeries, burn area, and donor site area.

**Results:**

Preoperative anxiety (NRS 4.86 ± 2.76) and fear (2.90 ± 2.50) decreased significantly after grafting (anxiety: 3.03 ± 2.48, *p* < 0.0001; fear: 2.21 ± 2.07, *p* < 0.0001). Multivariate analysis identified facial/hand burns (*β* = 0.18, *p* = 0.003) and multiple surgeries (*β* = 0.12, *p* = 0.028) as independent predictors of postoperative anxiety. Postoperative pain was common (56.6%) and correlated with residual anxiety (*r* = 0.42, *p* < 0.01). Patients receiving dual support (family plus clinician education) had significantly lower anxiety levels than those with non-dual support (3.92 ± 2.11 vs. 5.74 ± 2.83, *p* < 0.001). Prominent preoperative knowledge gaps (e.g., regarding graft failure risk) showed substantial postoperative improvement (*p* < 0.001).

**Conclusion:**

Preoperative anxiety and fear are prevalent but modifiable. The identification of independent predictors enables risk stratification for psychological support. The correlation between pain and anxiety and the benefit of dual support highlight the need for integrated, multidisciplinary care. These findings support embedding structured education and support systems into burn care pathways. Future prospective trials are warranted.

## 1 Introduction

Burns represent a form of accidental trauma involving damage to the skin, mucous membranes, muscles, bones, and internal organs caused primarily by thermal injury (1). Globally, burns cause over 180,000 deaths annually, with low-middle-income countries bearing 90% of the burden (2). In China, an estimated 10 million burn injuries occur yearly, of which 30–40% require grafting (3, 4). Beyond immediate physiological pain, burns often entail prolonged treatment and rehabilitation, imposing substantial medical costs and long-term sequelae such as scarring, disfigurement, and functional impairments (5). These factors contribute to post-traumatic stress disorder (PTSD) and persistent psychological distress, severely compromising patients’ quality of life and social functioning (6).

Early excision and skin grafting are critical for reducing pain, shortening treatment duration, and accelerating wound healing (7). Despite advances in analgesic techniques and surgical safety, patients frequently experience intense fear and anxiety before skin grafting surgery (8). This anticipatory distress peaks shortly before the procedure (9), triggering neuroendocrine responses (e.g., catecholamine release) that elevate blood pressure and heart rate, potentially compromising surgical outcomes and postoperative recovery (10). Consequently, psychological support for burn patients requiring skin grafting is essential for preventing psychosomatic complications.

Previous studies (11–13) identify multiple factors influencing burn patients’ mental health, including gender, age, education level, family environment, and burn severity. Proactive management of these factors during the perioperative period may enhance holistic recovery (14). However, regional disparities in healthcare infrastructure and patient demographics have hindered the development and implementation of standardized clinical guidelines, such as the NICE Clinical Guideline NG193 on burn rehabilitation (15), which recommends preoperative psychological assessment and structured communication. Moreover, existing research inadequately addresses the perioperative psychological state of burn patients or their perceptions of skin grafting (16). The conceptual link between ‘perceptions’ (encompassing knowledge, expectations, and emotions) and mental health lies in cognitive-behavioral theory: inaccurate beliefs (e.g., overestimating graft failure risk) can activate negative emotions (anxiety/fear), while accurate knowledge and positive expectations may buffer stress (9).

Prior studies also indicate 60–80% of grafting candidates experience clinically significant anxiety (8, 15), yet Chinese data remain scarce. International reports suggest cultural factors (e.g., stoicism in Asian patients) may mask distress (16), necessitating population-specific investigations. Our study fills this gap by quantifying both psychological changes and knowledge gaps in a Chinese cohort. This study also specifically focuses on burn patients undergoing skin grafting—a subgroup requiring repeated surgical interventions who face unique psychological challenges due to graft-related anxieties about outcomes that distinguish them from general burn populations. Compared to general burn patients, those undergoing skin grafting face distinct stressors: (1) invasive procedures involve both graft and donor sites, doubling physical trauma; (2) concerns about graft survival and long-term scarring on visible areas (face/hands) exacerbate social anxiety (5); (3) repeated surgeries (29.3% of our cohort underwent >2 grafts) create cumulative psychological burden. These factors distinguish their psychological needs from patients with superficial burns managed conservatively. We therefore aimed to characterize patients’ perceptions (knowledge, expectations, emotional responses) of skin grafting and quantify their relationship with mental health outcomes to inform targeted interventions. The primary objectives of this study were: (1) to quantify changes in anxiety, fear, and knowledge about skin grafting before and after surgery; (2) to identify modifiable factors (e.g., education, support systems) influencing these perceptions. Secondary objectives included: (1) examining correlations between pain and psychological outcomes; (2) exploring location-specific differences.

## 2 Methods

### 2.1 Study design, period, and area

This was a single-center retrospective cohort study. Data were collected retrospectively from the electronic medical records and standardized patient survey forms of Ningbo No. 2 Hospital, spanning January 2018 to December 2021. As part of the institution’s standard clinical care pathway, all eligible patients scheduled for skin grafting routinely complete self-report surveys at two time points: preoperatively (1–3 days before surgery) and postoperatively (at discharge). For this study, we extracted and analyzed these pre-existing, paired survey responses from the hospital’s database. This design allows for the analysis of within-subject changes over time within a retrospectively defined cohort. The study was reported in accordance with STROBE guidelines for observational studies (17) and followed the STROBE checklist (provided as Supplementary File 1).

This retrospective clinical study was approved by the Human Research Ethics Committee of Ningbo No.2 Hospital. Given the retrospective nature of the study and the use of anonymized data extracted from routine care records, the ethics committee granted a waiver of individual informed consent. The approval number was SL-NBEY-KY-2022-106-01.

### 2.2 Source and study population

**Figure 1:**
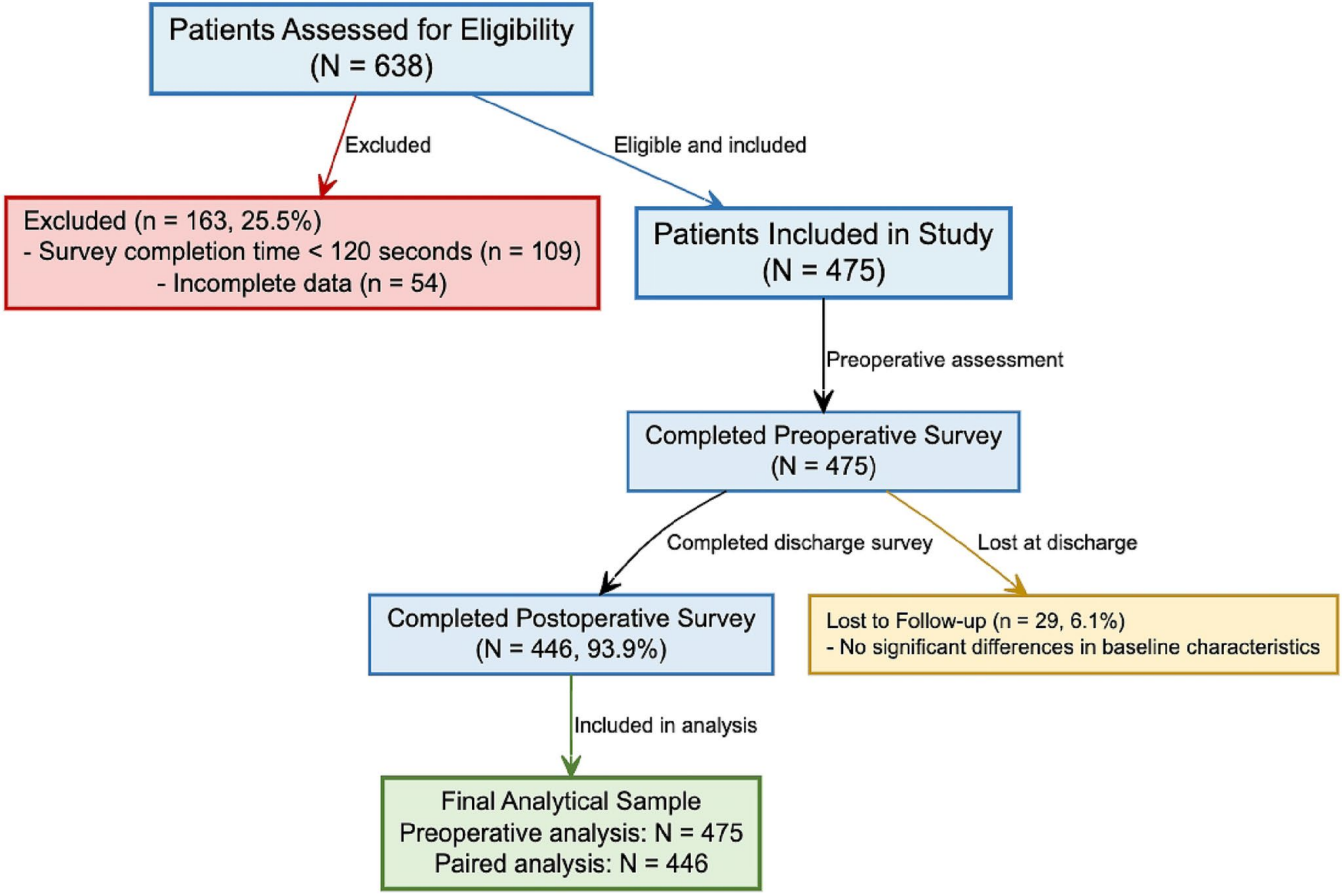
Participant flow diagram. This diagram illustrates the screening, enrollment, and follow-up process for the retrospective before-after study of burn patients’ perceptions of skin grafting. A total of 638 patients were assessed for eligibility at Ningbo No. 2 Hospital between January 2018 and December 2021. After exclusions (survey completion time <120 s and incomplete data), 475 patients (74.5%) were included. All included patients completed the preoperative survey. At discharge, 446 patients (93.9% of those included) completed the postoperative survey, forming the paired analysis sample. Twenty-nine patients (6.1%) were lost to follow-up at discharge, with no significant differences in baseline characteristics compared to those who completed the follow-up.

The study population consisted of all consecutively admitted adult burn inpatients at the Burn Department of Ningbo No. 2 Hospital during the study period who met the eligibility criteria and had completed the paired pre- and post-operative surveys as part of their clinical record. All survey content is presented in Chinese. All consecutively admitted burn inpatients were screened. Only those meeting grafting eligibility criteria (Section 2.3) and with completed survey data in their records were included. All non-graft burn patients (e.g., those with superficial burns managed by dressings alone) were excluded during initial screening, ensuring the cohort solely comprises individuals facing graft-specific psychological challenges. The screening and enrollment process is summarized in the participant flow diagram (Figure 1). Survey instruments assessing side effects and comfort-improving factors are detailed in the Supplementary methods (see Supplementary File 2).

### 2.3 Eligibility criteria

Inclusion: (1) ≥ 18 years; (2) underwent skin grafting during the index admission; (3) able to complete surveys (no severe cognitive impairment). Crucially, patients must have had both preoperative and postoperative survey data available in their medical records for inclusion in the paired analysis.

Exclusion: (1) non-graft burn patients; (2) history of psychiatric disorders (e.g., schizophrenia); (3) unable to read Chinese. (4) Medical records with missing preoperative or postoperative survey data.

### 2.4 Variables of the study

Data on study variables were extracted from the standardized survey forms stored in the electronic medical records. The survey included several screening questions to verify inclusion and exclusion criteria. The primary outcome measures were changes in anxiety and fear scores (using NRS) from pre- to post-surgery. Secondary outcomes included changes in HADS scores, perception scores (knowledge, expectations, emotions), and the prevalence of postoperative pain and its correlation with anxiety.

#### 2.4.1 Dependent variables

Patients’ perioperative psychological state was the primary focus, operationalized through: (1) Anxiety and fear perception scores measured via the Numerical Rating Scale (NRS); (2) Anxiety and depression subscale scores from the Hospital Anxiety and Depression Scale (HADS); (3) A composite ‘perception score’ reflecting understanding and views of skin grafting, derived from 10 structured items.

#### 2.4.2 Independent variables

Socio-demographic factors: age of the patient, sex, number of operations requiring skin grafting, etc.

Baseline information of disease: burn site, burn area, and burn depth, and site and area of donor site, etc.

Independent variables also included ‘dual support’ (defined as receiving both family emotional support and structured clinician education) and ‘non-dual support’ (receiving only one type of support or none), as documented in nursing notes and survey responses.

#### 2.4.3 Standard preoperative education

Standard preoperative education was delivered by attending surgeons (15-min consultation) and anesthesiologists (10-min discussion) 2–3 days before surgery, as documented in the nursing and medical notes from which this variable was extracted. Content included: (1) graft type (split-thickness/full-thickness), (2) surgical steps (donor site harvesting, graft application), (3) risks (e.g., 5–8% failure rate), (4) anesthesia side effects, and (5) printed FAQ leaflets (with diagrams) reviewed by nurses on the day before surgery.

### 2.5 Operational definitions

“Perceptions” in this study were operationally defined and measured through three complementary approaches: (a) Intensity of anxiety and fear using a self-rated 0–10 Numerical Rating Scale (NRS) [NEW REF for NRS; suggested by Reviewer 2]; (b) Screening for clinically significant anxiety and depression symptoms using the validated Hospital Anxiety and Depression Scale (HADS) (14, 15); (c) Content-specific perceptions assessed via structured questions on knowledge (5 items, correct/incorrect), expectations (3 items, positive/negative), and emotional responses (2 items, positive/negative), which were summed to create a total perception score (range 0–10, higher scores indicating better understanding/more positive perceptions).

“Understanding” referred specifically to the knowledge domain and was assessed as a binary (Yes/No) response to 5 key factual questions about the grafting process (e.g., “Skin grafts require donor sites”).

### 2.6 Sample size and participant flow

A total of 638 patient records were initially assessed for eligibility during the study period (January 2018 to December 2021). Of these, 475 records (74.5%) met all inclusion criteria and were included in the study. A total of 163 records (25.5%) were excluded: 109 (17.1% of the initial 638) because the survey completion time was recorded as <120 s (suggesting potentially inattentive responding), and 54 (8.5%) due to incomplete data (e.g., missing preoperative or postoperative scores).

This analysis utilized a within-subjects paired design on the retrospectively identified cohort. All 475 included patients had preoperative survey data. Postoperative survey data at discharge (median 14 days) were available for 446 (93.9%) of these patients, forming the paired sample for pre-post comparisons. Data were matched using unique hospital IDs, ensuring preoperative and postoperative data originated from the same individual. Postoperative survey data at discharge were available for 446 of the 475 included patients (93.9%). Twenty-nine patients (6.1%) were lost to follow-up at discharge (i.e., missing postoperative survey data).

### 2.7 Data source and collection procedure

Data for this study were sourced exclusively from the electronic medical records of Ningbo No. 2 Hospital. As per hospital protocol, multiple physicians and nurses from the burn department were responsible for administering standardized paper surveys to eligible patients during their routine clinical care at the predefined timepoints (preoperatively, 1–3 days before surgery, and postoperatively, at discharge). Completed surveys were then scanned and stored in the patients’ electronic health records. For this retrospective study, data from these stored survey forms were systematically extracted into a structured database for analysis. Our aim was to understand patients’ opinions, concerns, fears, and beliefs about skin grafting before and after surgery, using this routinely collected data.

The survey questionnaire (Supplementary File 2) included: (1) 5 eligibility screening questions (e.g., ‘Will you undergo skin grafting within 1 month?’); (2) 8 perception questions (e.g., ‘Do you know the graft may feel numb temporarily?’); (3) Numerical Rating Scale (NRS, 0–10) items for anxiety and fear; (4) Hospital Anxiety and Depression Scale (HADS) items (e.g., ‘I feel tense or wound up’). In the process of collecting patients’ anxiety and fear as part of routine care, both patient self-evaluation (using NRS) and staff-administered evaluation (using HADS) were performed. NRS was used to record anxiety and fear scores, while HADS was used to record anxiety and depression subscale scores. The NRS uses a 0–10 point scale (0 = no anxiety/fear, 10 = extreme anxiety/fear). The HADS-Anxiety and HADS-Depression subscales each range from 0 to 21, with scores ≥8 indicating clinical significance (14). Both the NRS and HADS have been validated in Chinese surgical populations. In this study, the internal consistency (Cronbach’s *α*) was 0.82 for the NRS items and 0.78 for the HADS, indicating acceptable reliability. The staff-administered HADS evaluation was carried out by an experienced nurse. STROBE guidelines for observational studies were followed in the reporting of this study (17).

### 2.8 Data quality management

To ensure data quality and address potential biases, multiple measures were implemented. Survey completeness and accuracy in the source records were verified by the research investigators during the data extraction process. To mitigate self-reporting bias, both self-rated (NRS) and staff-administered (HADS) measures were employed in the original data collection. To minimize social desirability bias in the retrospectively used data, nursing staff were trained to administer surveys in a neutral manner as part of standard clinical practice. Data quality for the research analysis was further ensured by excluding responses completed within 120 s (as recorded in the database) and incomplete surveys during the data extraction phase. Missing data were minimal (<2%) and handled using pairwise deletion for the respective analyses.

### 2.9 Data processing and analysis procedures

We conducted both univariate and multivariate analyses. For within-subject comparisons, continuous variables were analyzed using paired t-tests (for normally distributed data) or Wilcoxon signed-rank tests (for non-normal distributions), while categorical variables were assessed with McNemar’s test. Effect sizes for pre-post changes were calculated as Cohen’s d for continuous outcomes. To control for potential confounders (age, sex, burn site, number of surgeries, burn area, and donor site area), we performed multiple linear regression analyses with postoperative anxiety (NRS) as the dependent variable. All statistical tests were two-tailed with significance set at *p* < 0.05, and 95% confidence intervals are reported for correlation and regression coefficients. Data were analyzed using Microsoft Excel and SPSS version 26.0.

For the multiple linear regression analysis, the model was constructed as follows:


$$\begin{array}{c} \text{Postoperative Anxiety}\;(\mathrm{NRS})=\beta_{0}+\beta_{1}\times (\mathrm{Age})+\beta_{2}\times (\mathrm{Sex})\\ +\beta_{3}\times (\text{Facial}/\text{Hand Burn})\\ +\beta_{4}\times (\text{Number of Surgeries})\\ +\beta_{5}\times (\text{Burn Area}\%\text{TBSA})\\ +\beta_{6}\times (\text{Donor Site Area})+\varepsilon \end{array}$$

Model assumptions were checked, including linearity, homoscedasticity, and normality of residuals, with no major violations detected. Variance Inflation Factors (VIF) for all predictors were below 2.0, indicating no concerning multicollinearity.

## 3 Results

### 3.1 Participant flow and analytical samples

Following the screening process detailed in Figure 1, a total of 475 patients were included in the study with complete preoperative data. Of these, 446 patients (93.9%) completed the postoperative survey at discharge, forming the paired sample for pre-post comparisons. Therefore, the analytical sample comprised *N* = 475 for preoperative analyses and *N* = 446 for paired analyses (Figure 1).

### 3.2 Socio-demographic characteristics

The average age of the respondents was 42 (35, 48) years, including 289 males (60.8%) and 186 females (39.2%), all of whom all were cisgender (100%). Detailed baseline characteristics are presented in Table 1. We examined the association between baseline characteristics and preoperative anxiety (NRS). Significant correlations were found between preoperative anxiety and burn site (facial/hand vs. trunk, *r* = 0.21, *p* < 0.001), number of surgeries (r = 0.15, *p* = 0.001), and burn area (*r* = 0.13, *p* = 0.006). No significant associations were found with age, sex, or BMI (*p* > 0.05).

**Table 1:** Demographic characteristics (*N* = 475).

Variable	Subgroup	*N* = 475
Age, years		42 (35, 48)
Sex	Male	289 (60.8%)
	Female	186 (39.2%)
Height (m)		1.67 (1.57, 1.73)
Weight (kg)		69.78 ± 13.79
BMI, kg/m^2^		23.53 ± 3.53
Drinking history	Has	269 (56.6%)
	Has not	206 (43.4%)
Smoking history	Has	316 (66.5%)
	Has not	159 (33.5%)
Disease diagnosis	Hypertension	28 (5.9%)
	Type II diabetes mellitus	19 (4.0%)
	Others	36 (7.6%)
Major burn site	More than one item of head, face, neck	21 (4.4%)
	Upper and/or lower limbs	387 (81.5%)
	Trunk	54 (11.4%)
	Buttocks	13 (2.7%)
	Perineum	0 (0%)
Major donor site	Upper and/or lower limbs	446 (93.9%)
	Trunk	29 (6.1%)
Donor area (%TBSA)	1–10%	357 (75.2%)
	10–20%	69 (14.5%)
	>20	38 (8.0%)
Burn surface area (%TBSA)	1–10%	50 (10.5%)
	10–20%	162 (34.1%)
	20–30%	174 (36.6%)
	>30%	89 (18.7%)
Burn depth	Partial Thickness Burn	319 (67.2%)
	Full-thickness Burn	156 (32.8%)
Numbers of skin grafting	1	249 (52.4%)
	2	87 (18.3%)
	>2	139 (29.3%)

### 3.3 Role of others’ influence on participant thoughts and opinions towards skin grafting

Among 340 respondents, 289 (85.0%) reported positive influences (family members with prior surgery: 56/289; medical staff: 298/340). Negative influences occurred in 51 (15.0%). Patients receiving dual support (family+clinician, *n* = 267) had significantly lower preoperative anxiety (NRS 3.92 ± 2.11) compared to those with non-dual support (*n* = 208, NRS 5.74 ± 2.83, *p* < 0.001).

### 3.4 Comprehensive assessment of skin graft perceptions and psychological outcomes

Before undergoing skin grafting, patients reported moderate preoperative anxiety (NRS score: 4.86 ± 2.76) and fear (NRS: 2.90 ± 2.50). A structured assessment of perceptions revealed significant gaps and mismatches (detailed data in Supplementary Table S1). Key findings included: (1) Knowledge gaps: Only 28.6% were aware of graft failure risk. (2) Expectation mismatches: 68.2% expected perfect color match. (3) Emotional burden: 33.3% expressed feelings of hopelessness.

Postoperatively, significant improvements were observed. Anxiety and fear levels decreased substantially (anxiety: 3.03 ± 2.48, *p* < 0.0001; fear: 2.21 ± 2.07, *p* < 0.0001). Postoperative HADS scores also improved (anxiety: 5.72 ± 2.19 vs. 4.32 ± 1.94; depression: 2.44 ± 1.49 vs. 1.74 ± 1.07; both *p* < 0.0001) (Table 2). The proportion of patients considering surgery cancellation decreased from 7.2% preoperatively to 3.2% postoperatively (*p* < 0.001). Notably, patients with facial or hand burns maintained higher residual anxiety compared to those with trunk burns (3.98 ± 2.33 vs. 2.41 ± 1.89, *p* = 0.002). Postoperative pain, reported by 56.6% of patients, showed a moderate correlation with residual anxiety (*r* = 0.42, 95% CI 0.35–0.49, *p* < 0.01) (Table 3).

**Table 2:** Anxiety scores and fear scores before and after skin grafting.

Before and after skin grafting	Numbers	Anxiety scores (NRS)	Fear scores (NRS)	Anxiety scores (HAD)	Depression scores (HAD)
Before skin grafting	453	4.86 ± 2.76	2.90 ± 2.50	5.72 ± 2.19	2.44 ± 1.49
After skin grafting	446	3.03 ± 2.48	2.21 ± 2.07	4.32 ± 1.94	1.74 ± 1.07
Value of change	–	1.83	0.69	1.41	0.70
*P* Value	–	<0.0001	<0.0001	<0.0001	<0.0001

**Table 3:** Multidimensional perception assessment with effect sizes (*N* = 475).

Perception domain and specific items	Preoperative positive response	Postoperative positive response	*p*-value	Effect size (Cohen’s d)
Knowledge				
Knows donor site heals in 2–3 weeks	42.1% (200)	89.5% (425)	<0.001	1.12
Understands graft numbness risk	33.7% (160)	75.8% (360)	<0.001	0.98
Aware of graft failure risk (5–8%)	28.6% (136)	92.0% (437)	<0.001	1.84
Expectations				
Expects perfect color match	68.2% (324)	21.3% (101)	<0.001	−1.05
Anticipates immediate functionality	51.8% (246)	12.6% (60)	<0.001	−0.92
Believes no further surgeries needed	39.6% (188)	63.4% (301)	<0.001	0.49
Emotions				
Feels hopeful about outcome	35.6% (169)	72.6% (345)	<0.001	0.82
Reports no hopelessness	41.9% (199)	84.2% (400)	<0.001	0.96
Denies extreme fear of disfigurement	45.9% (218)	81.7% (388)	<0.001	0.78
Behavioral intention				
Would not consider future surgery cancellation	92.8% (441)	96.8% (460)	<0.001	0.45

To control for potential confounders, we performed a multiple linear regression analysis with postoperative anxiety (NRS) as the outcome (Table 4). The model was statistically significant (R^2^ = 0.24, *F*(6, 439) = 9.87, *p* < 0.001). After adjustment, facial/hand burns (*β* = 0.18, 95% CI [0.06, 0.30], *p* = 0.003) and number of surgeries (*β* = 0.12, 95% CI [0.01, 0.23], *p* = 0.028) remained significant independent predictors of higher postoperative anxiety. Age, sex, burn area, and donor site area were not significant predictors in the final model (all *p* > 0.05).

**Table 4:** Multiple linear regression analysis of factors associated with postoperative anxiety (NRS).

Predictor variable	β coefficient	95% CI for β	Standard error	Standardized β	t-value	*p*-value
(Intercept)	2.15	[1.08, 3.22]	0.54	-	3.98	<0.001
Age (years)	−0.01	[−0.03, 0.01]	0.01	−0.04	−0.87	0.385
Sex (Male)	0.18	[−0.15, 0.51]	0.17	0.04	1.06	0.290
Facial/hand burns (Yes)	0.68	[0.24, 1.12]	0.22	0.18	3.02	0.003
Number of surgeries	0.21	[0.02, 0.40]	0.10	0.12	2.20	0.028
Burn area (%TBSA)	0.01	[−0.01, 0.03]	0.01	0.06	1.31	0.190
Donor site area	0.02	[−0.02, 0.06]	0.02	0.05	1.01	0.314

Model Summary: *R*^2^ = 0.24, Adjusted *R*^2^ = 0.23, F(6, 439) = 9.87, *p* < 0.001. The intercept represents the predicted baseline postoperative anxiety score (on a 0–10 NRS) for a theoretical reference patient with values of zero for all predictor variables (e.g., no facial/hand burns, zero prior surgeries).

### 3.5 Participants’ perceived side effects from skin grafting

**Figure 2:**
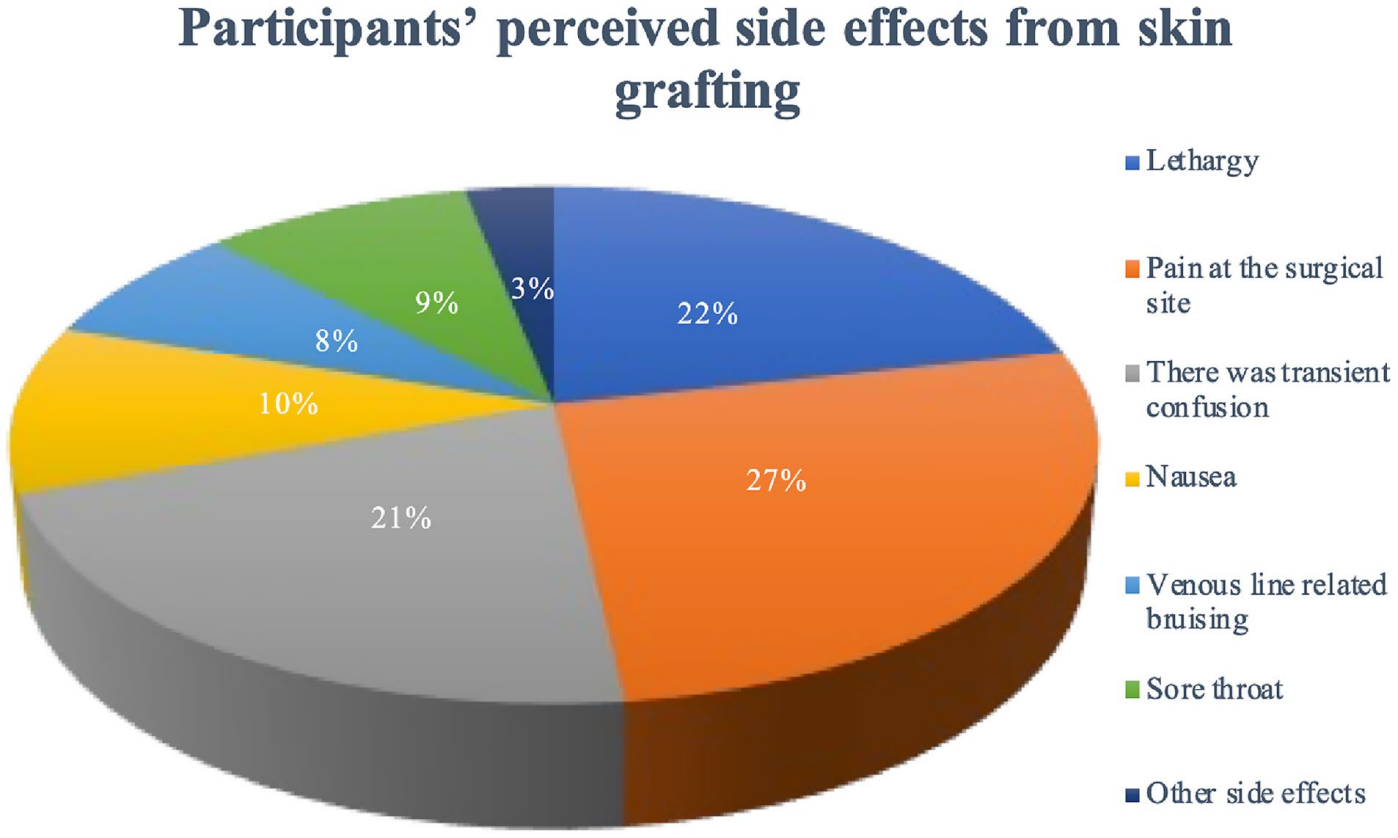
Participants’ perceived side effects from skin grafting (*N* = 475). Data show the distribution of commonly reported side effects following skin grafting procedures.

Survey participants were asked to self-report side effects following skin grafting. In the short term (1 day after surgery), most patients had mild adverse reactions (86.1%), mainly drowsiness (45.9%), surgical site pain (56.6%), and transient confusion (45.3%). The distribution of common side effects is summarized in Figure 2; a detailed breakdown is provided in Supplementary Table S2. Postoperative pain (56.6%) showed a moderate correlation with residual anxiety (*r* = 0.42, *p* < 0.01), while other side effects (e.g., drowsiness, nausea) had no significant association (*p* > 0.05). This suggests pain management is critical for reducing post-graft anxiety.

### 3.6 What participants believed would improve their comfort level towards skin grafting

**Figure 3:**
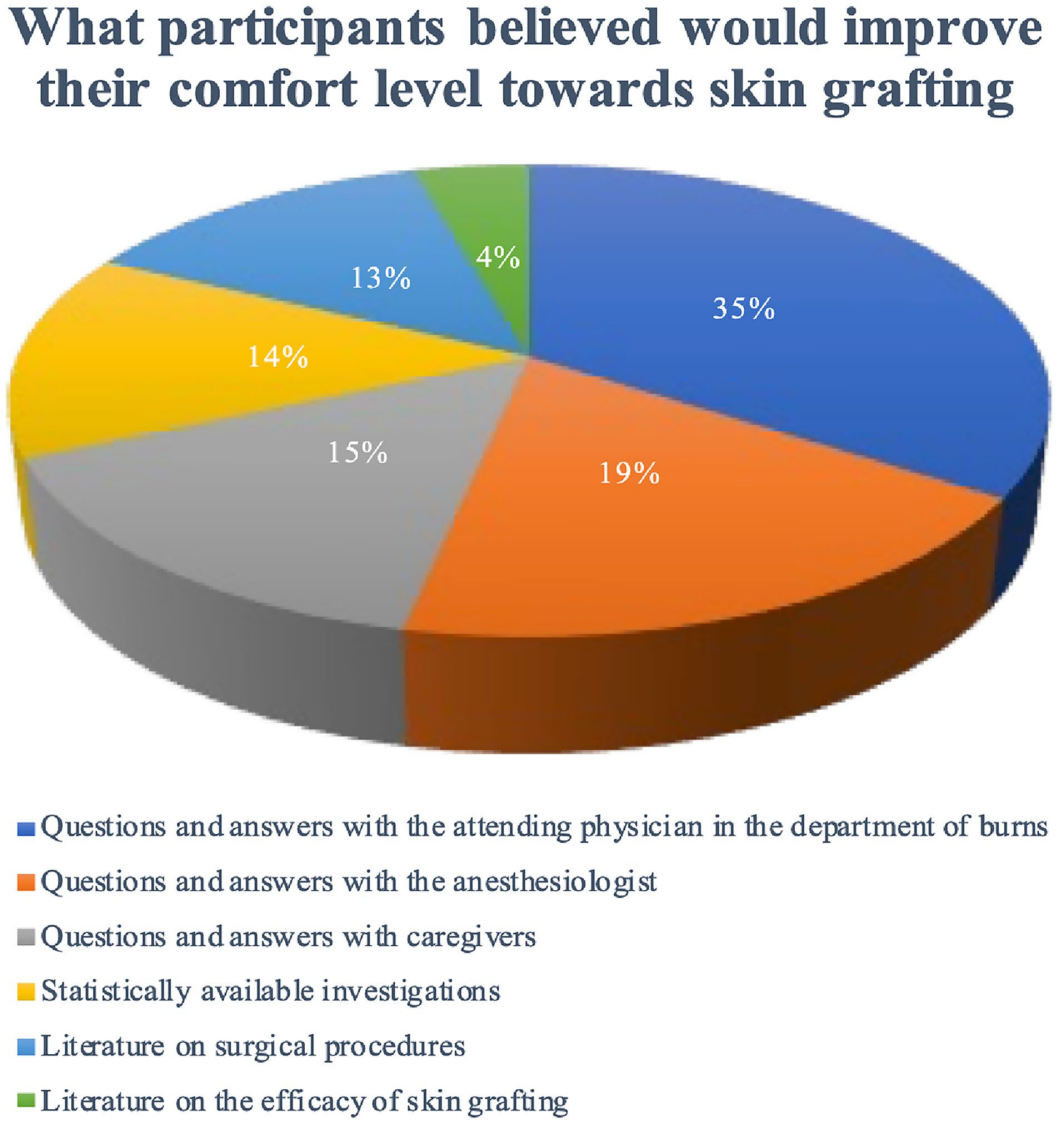
Interventions believed to improve comfort with skin grafting (*N* = 475). Patient preferences for various comfort-enhancing interventions are displayed.

Respondents were asked what interventions would improve their comfort. The most frequently requested were discussions with the attending burn physician (97.7%) and with the anesthesiologist (53.3%). Patient preferences are summarized in Figure 3 (detailed data in Supplementary Table S3). Crucially, patients who received consultations with both physician and anesthesiologist (*n* = 248) reported 32% lower postoperative anxiety than those receiving single consultations (*p* = 0.004).

## 4 Discussion

Our retrospective cohort study provides novel insights into the psychological trajectory of Chinese burn patients undergoing skin grafting, revealing three key advances in understanding their perioperative experiences. Key findings include: (1) Significant but modifiable preoperative knowledge gaps and emotional distress; (2) The identification of facial/hand burns and multiple surgeries as independent predictors of postoperative anxiety; (3) A strong correlation between postoperative pain and anxiety; (4) A substantial psychological benefit associated with receiving combined family and clinician support (‘dual support’). These findings extend Von Korff et al.’s cognitive-behavioral model (18) by demonstrating how inaccurate medical knowledge activates threat appraisal systems in a Chinese clinical context. The differential reduction in anxiety (37.7%) versus fear (23.8%) post-grafting supports neurocognitive models where procedural familiarity selectively modulates pain perception pathways (19). Our multivariate analysis identified facial/hand burns and multiple surgeries as independent predictors of persistent anxiety, reflecting activation of social threat networks in limbic regions (20) that necessitate targeted interventions beyond standard care.

Our findings demonstrate a significant correlation between postoperative pain and residual anxiety (*r* = 0.42, *p* < 0.01), with 56.6% of participants reporting surgical site pain. This extends established literature by quantifying the pain-anxiety relationship specifically in skin grafting patients, showing that uncontrolled pain activates the limbic system’s social threat network (16) in this vulnerable population. The maintained higher anxiety levels in facial/hand burn patients (NRS 3.98 ± 2.33 vs. trunk burns 2.41 ± 1.89, *p* = 0.002) likely reflects heightened concerns about social visibility and disfigurement, further supporting this mechanism. These observations are consistent with cognitive-behavioral models where pain and threat perception share neural pathways (16, 17). Neuroimaging evidence from Darvish-Ghane et al. provides mechanistic insight: concurrent activation of the anterior cingulate cortex and insula during pain-anxiety episodes creates a self-perpetuating cycle where pain triggers emotional distress through shared pathways, while anxiety amplifies pain perception via catecholamine release (10). Our clinical data validate this bidirectional relationship in burn grafting patients, underscoring the necessity of integrating multimodal analgesia—combining pharmacologic interventions with non-pharmacologic approaches like virtual reality therapy—to disrupt this cycle.

Our behavioral findings reveal that surgery cancellation consideration decreased significantly postoperatively (7.2 to 3.2%, *p* < 0.001), coinciding with major improvements in knowledge accuracy. For instance, awareness of graft failure risk increased from 28.6 to 92.0%. This suggests that correcting specific knowledge gaps, rather than general reassurance, may be a key driver of improved psychological adaptation and treatment adherence.

The observational design precludes definitive causal inferences—for example, anxiety reduction may reflect natural recovery trajectories alongside procedural familiarity. However, the identification of independent predictors through multivariate analysis strengthens the argument for targeted interventions. The consistency of our core findings (e.g., high preoperative anxiety, benefit of education) with international studies (8, 17, 19) suggests that enhanced, structured clinician-patient communication protocols warrant further investigation through randomized trials.

The influence of social and clinical support was pronounced: patients receiving dual support (family + clinician education) showed 32% lower anxiety levels, supporting Brown et al.’s concept that coherent support systems provide ‘social safety signals’ that buffer surgical stress (20) while demonstrating its cross-cultural applicability in a Chinese healthcare setting. Transparent risk communication combined with realistic expectation calibration emerged as a critical factor in reducing cancellation consideration, challenging conventional approaches that might avoid discussing complications and proving associated with reduced cancellation rates without increasing distress. The influence of social networks on patient perceptions warrants particular attention: while most participants received reassuring feedback from acquaintances with grafting experience, negative anecdotes significantly heightened concern—particularly regarding graft necrosis and scar hyperplasia. This social amplification effect was most pronounced in patients with facial/hand burns, where social visibility amplified anxiety, highlighting the need for structured peer-support programs that leverage positive social influence while mitigating misinformation.

Our data translate into three specific, evidence-based recommendations for clinical practice: (1) Structured preoperative education should prioritize correcting the critical knowledge gaps identified in our cohort (e.g., donor site healing timelines, sensory changes, graft survival rates), as detailed in Supplementary Table S1, moving beyond general reassurance; (2) Clinical protocols should formally facilitate “dual support” by combining structured family engagement with clinician counseling, given its association with significantly lower anxiety levels; (3) For patients with facial or hand burns—identified as an independent predictor of anxiety—tailored interventions addressing social threat sensitivity (e.g., through preparatory counseling focusing on appearance and function) should be developed.

These recommendations align with and refine existing calls for enhanced communication (21) and early psychological intervention (22–24) by pinpointing the specific content (knowledge gaps) and target populations (those with visible burns or lacking dual support) for such efforts. Since burn patients may not proactively express distress, our findings underscore the necessity for healthcare providers to initiate these proactive, structured interventions, particularly for the high-risk subgroups identified.

Furthermore, the strong correlation between postoperative pain and anxiety (*r* = 0.42) necessitates integrated pain and psychological management. While common anesthetic side effects are often self-limiting (25–27), unmanaged surgical pain appears to be a key modifiable contributor to psychological morbidity. Our findings therefore advocate for the integration of validated non-pharmacologic adjuncts [e.g., virtual reality therapy (28)] within multimodal analgesia protocols to address this pain-anxiety cycle. A comprehensive, multimodal approach to rehabilitation—encompassing physical, psychological, and social components—is supported by our data and remains essential for optimal recovery (29–34).

### 4.1 Clinical practice implications

Drawing directly from our findings, we propose several key implications for clinical practice, structured around the core themes of targeted intervention, integrated care, and continuity of support.

#### 4.1.1 Towards multidisciplinary, integrated care

Our identification of facial/hand burns and multiple surgeries as independent predictors of anxiety, coupled with the strong pain-anxiety correlation, underscores that psychological morbidity is multifactorial. This supports a structured, multidisciplinary approach. Embedding psychological assessment and support early in the care pathway can address anticipatory anxiety. Concurrently, dedicated pain management protocols are crucial, as unmanaged pain appears to be a key, modifiable driver of distress. Postoperatively, rehabilitation must address both physical function and psychological barriers like kinesiophobia (35, 36). A coordinated team—integrating surgeons, anesthesiologists, nurses, psychologists, pain specialists, and therapists—is needed to address this biopsychosocial complexity holistically.

#### 4.1.2 Ensuring continuity of care beyond discharge

The significant prevalence of postoperative pain (56.6%) and psychological vulnerability at discharge highlights a critical gap in transitional care. Community-based healthcare providers, especially nurses, are essential in bridging this gap. Through home visits or structured telemedicine follow-up (37, 38), they can monitor recovery, manage wounds, and—vitally—screen for emerging anxiety or depression, facilitating timely referral. This continuous, patient-centered support fosters adherence and empowers patients, potentially improving long-term outcomes.

#### 4.1.3 Standardizing proactive education and psychological screening

The variability in patient knowledge and outcomes calls for standardized protocols. Preoperative education must be proactive and structured, specifically targeting the prevalent knowledge gaps we identified (e.g., graft survival, donor site healing), which is associated with reduced surgery cancellations. Postoperatively, routine psychological screening using validated tools (e.g., HADS) (39) at discharge and follow-up can systematically identify patients requiring further mental health support. Formalizing these elements into a comprehensive program, akin to the ‘dual support’ model that benefited our cohort, can enhance preparedness, reduce anxiety, and improve recovery trajectories.

### 4.2 Limitations

This study has limitations. Its single-center, retrospective design may limit generalizability to other settings (40), and social desirability bias may have led to underreporting of negative experiences. The 25.5% exclusion rate (primarily due to short survey completion time or missing data) may introduce participation bias. Assessment was at discharge (median 14 days), preventing conclusions about long-term psychological trajectories. Although we controlled for key confounders, unmeasured variables (e.g., socioeconomic status, pre-morbid resilience) and residual confounding remain possible, as indicated by the model’s explanatory power (R^2^ = 0.24). The predominantly male, culturally homogeneous sample also limits generalizability.

Despite these limitations, our study provides novel insights into the perioperative psychology of Chinese burn graft patients. The consistency of our core findings—regarding modifiable anxiety, key predictors, and the value of support—with international literature (41, 42) strengthens their validity. The limitations clearly outline the need for future prospective (43), multicenter studies (44) with long-term follow-up (45) and mixed-methods approaches (46, 47)to confirm and extend our findings.

## 5 Conclusion

This study demonstrates that preoperative anxiety and fear are prevalent but modifiable among burn patients undergoing skin grafting, with significant reductions achieved through targeted interventions. We identified facial/hand burns and multiple surgeries as independent predictors of postoperative anxiety, enabling evidence-based risk stratification. The observed strong correlation between pain and anxiety, coupled with the significant benefit associated with dual support systems, highlights the importance of integrated biopsychosocial care. While our observational data support the value of targeted interventions, future prospective studies are needed to establish causal efficacy. These findings advocate for embedding multidisciplinary teams—including psychologists, pain specialists, and rehabilitation therapists—into standard burn care pathways, with structured preoperative education and nurse-led psychological support serving as essential components for holistic patient recovery.

## Data Availability

The original contributions presented in the study are included in the article/Supplementary material, further inquiries can be directed to the corresponding authors.

## Supplementary material

SUPPLEMENTARY FIGURE 1 Participants' perceived side effects from skin grafting (*N* = 475). Data are presented as percentages of patients reporting each symptom. Surgical site pain (56.6%) showed a significant moderate correlation with residual anxiety (*r* = 0.42, *p* < 0.01), while other side effects showed no significant associations. One case of transient blindness was reported and resolved spontaneously. Error bars represent standard error.

SUPPLEMENTARY FIGURE 2 Interventions believed to improve comfort with skin grafting (*N* = 475). Direct communication with healthcare providers was the most frequently requested intervention. Patients who received both physician and anesthesiologist consultations (*n* = 248) reported 32% lower postoperative anxiety compared to those receiving single consultations (*p* = 0.004). Data are presented as percentages of patients requesting each intervention.
