# Supplementary figures and images for: Burn patients’ perceptions of skin grafting in China: a single-center retrospective cohort study with paired pre-post assessment
Source: Front Public Health. 2026 Jan 23;14:1754982. doi: 10.3389/fpubh.2026.1754982 (PMC12875900; doi:10.3389/fpubh.2026.1754982)

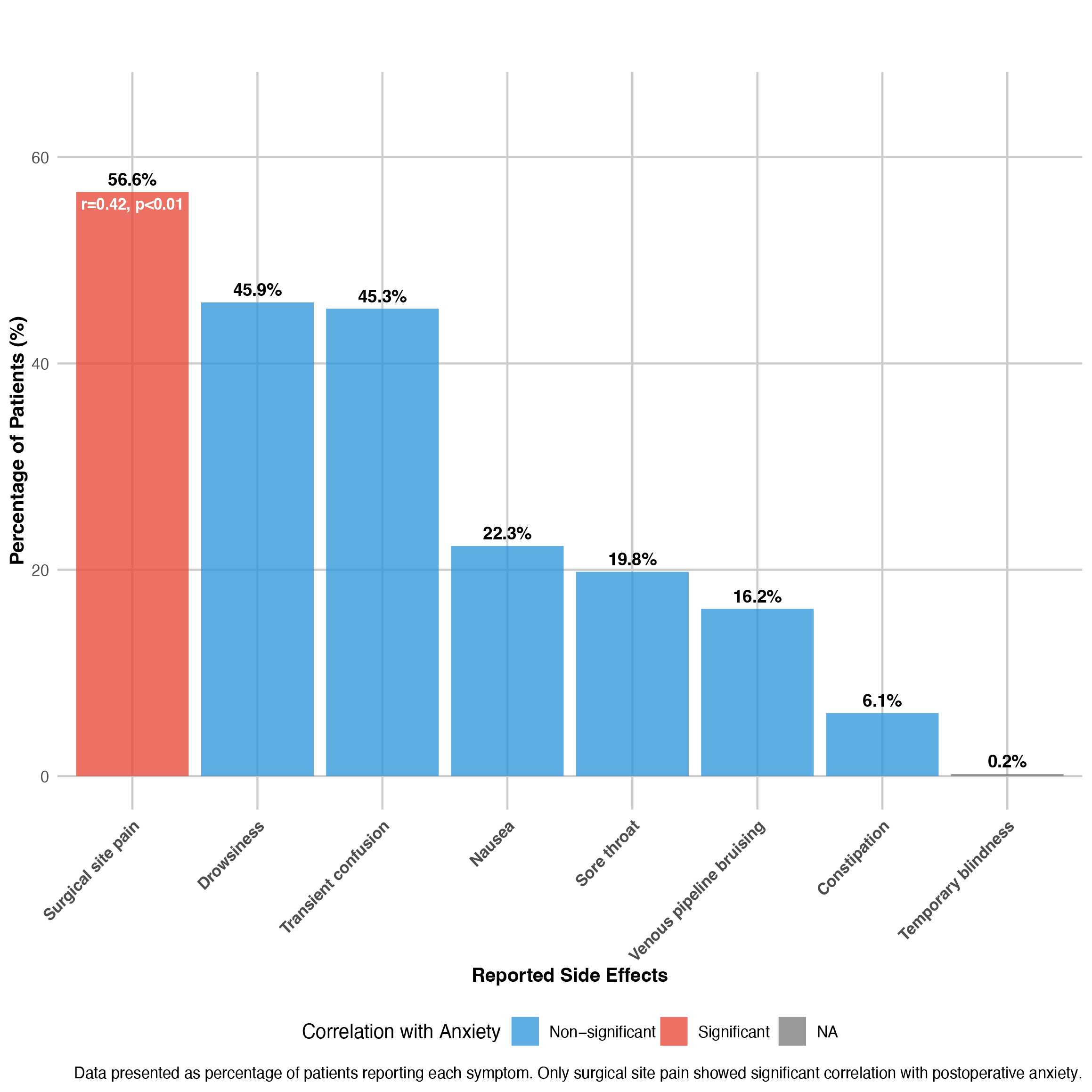

Supplement: SUPPLEMENTARY FIGURE 1 — Participants' perceived side effects from skin grafting (N = 475). Data are presented as percentages of patients reporting each symptom. Surgical site pain (56.6%) showed a significant moderate correlation with residual anxiety (r = 0.42, p < 0.01), while other side effects showed no significant associations. One case of transient blindness was reported and resolved spontaneously. Error bars represent standard error. [file Image_1.tif]

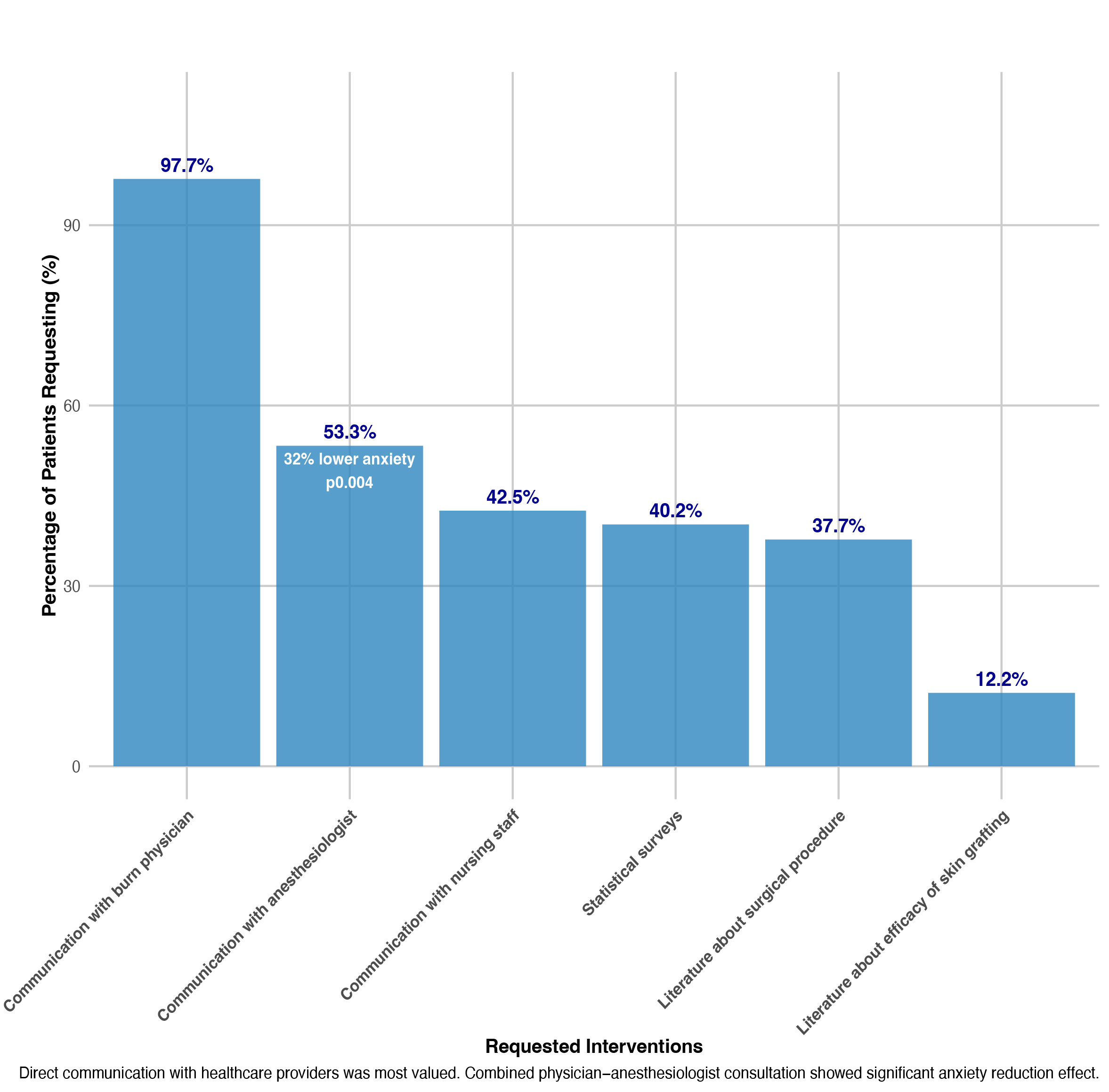

Supplement: SUPPLEMENTARY FIGURE 2 — Interventions believed to improve comfort with skin grafting (N = 475). Direct communication with healthcare providers was the most frequently requested intervention. Patients who received both physician and anesthesiologist consultations (n = 248) reported 32% lower postoperative anxiety compared to those receiving single consultations (p = 0.004). Data are presented as percentages of patients requesting each intervention. [file Image_2.tif]
