# Supplementary material for: Burn patients’ perceptions of skin grafting in China: a single-center retrospective cohort study with paired pre-post assessment
Source: Front Public Health. 2026 Jan 23;14:1754982. doi: 10.3389/fpubh.2026.1754982 (PMC12875900; doi:10.3389/fpubh.2026.1754982)
Supplement: Supplementary file 3 [file Table_1.docx]

**Supplementary Table S1. Detailed Pre- and Post-operative Assessment of Skin Graft Perceptions (N=475)**

| Perception Domain & Specific Item | Preoperative Positive Response N (%) | Postoperative Positive Response N (%) | P-value (McNemar's Test) | Effect Size (Cohen's d) |
| --- | --- | --- | --- | --- |
| **Knowledge** |  |  |  |  |
| Knows donor site heals in 2-3 weeks | 200 (42.1%) | 425 (89.5%) | <0.001 | 1.12 |
| Understands risk of temporary graft numbness | 160 (33.7%) | 360 (75.8%) | <0.001 | 0.98 |
| Aware of graft failure risk (5-8%) | 136 (28.6%) | 437 (92.0%) | <0.001 | 1.84 |
| Knows grafts require donor sites | 315 (66.3%) | 462 (97.3%) | <0.001 | 0.85 |
| Aware of potential for long-term scar contracture | 89 (18.7%) | 388 (81.7%) | <0.001 | 1.52 |
| **Expectations** |  |  |  |  |
| Expects perfect color match between graft and native skin | 324 (68.2%) | 101 (21.3%) | <0.001 | -1.05 |
| Anticipates immediate functional recovery post-grafting | 246 (51.8%) | 60 (12.6%) | <0.001 | -0.92 |
| Believes no further surgeries will be needed | 188 (39.6%) | 301 (63.4%) | <0.001 | 0.49 |
| **Emotional Response** |  |  |  |  |
| Feels hopeful about the surgical outcome | 169 (35.6%) | 345 (72.6%) | <0.001 | 0.82 |
| Reports no feelings of hopelessness | 199 (41.9%) | 400 (84.2%) | <0.001 | 0.96 |
| Denies extreme fear of disfigurement | 218 (45.9%) | 388 (81.7%) | <0.001 | 0.78 |
| **Behavioral Intention** |  |  |  |  |
| Would not consider cancelling a future surgery if needed | 441 (92.8%) | 460 (96.8%) | <0.001 | 0.45 |

*Note: “Positive Response” is defined as a correct answer for Knowledge items, a realistic/positive expectation for Expectation items, and the absence of a negative emotion or presence of a positive one for Emotional items. P-values and effect sizes (Cohen's d for proportions) are calculated for the within-subject change from pre- to post-operation.*
