# Supplementary material for: Burn patients’ perceptions of skin grafting in China: a single-center retrospective cohort study with paired pre-post assessment
Source: Front Public Health. 2026 Jan 23;14:1754982. doi: 10.3389/fpubh.2026.1754982 (PMC12875900; doi:10.3389/fpubh.2026.1754982)
Supplement: Supplementary file 4 [file Table_2.docx]

**Supplementary Table S2. Detailed Profile of Patient-Perceived Side Effects Following Skin Grafting (N=475)**

| **Side Effect (Short-term, within 1 day post-op)** | **Number of Patients Reporting (N)** | **Percentage (%)** | **Correlation with Post-op Anxiety (NRS) [r (p-value)]** |
| --- | --- | --- | --- |
| Any mild adverse reaction | 409 | 86.1% | -- |
| Surgical site pain | 269 | 56.6% | 0.42 (p<0.01) |
| Drowsiness | 218 | 45.9% | 0.05 (p=0.28) |
| Transient confusion | 215 | 45.3% | 0.03 (p=0.51) |
| Nausea | 106 | 22.3% | 0.07 (p=0.15) |
| Sore throat | 94 | 19.8% | 0.04 (p=0.39) |
| Venous pipeline related bruising | 77 | 16.2% | 0.02 (p=0.69) |
| **Other reported effects:** |  |  |  |
| Constipation* | 29 | 6.1% | 0.10 (p=0.06) |
| Transient blindness (resolved) | 1 | 0.2% | -- |

Note: *Constipation was the most frequently cited item under the "other" category. Correlation coefficients (Pearson's r) are calculated between the presence/severity of the side effect (where applicable) and the postoperative anxiety NRS score. Only surgical site pain showed a statistically significant (p<0.01) moderate positive correlation with residual anxiety.
