# Supplementary material for: Burn patients’ perceptions of skin grafting in China: a single-center retrospective cohort study with paired pre-post assessment
Source: Front Public Health. 2026 Jan 23;14:1754982. doi: 10.3389/fpubh.2026.1754982 (PMC12875900; doi:10.3389/fpubh.2026.1754982)
Supplement: Supplementary file 5 [file Table_3.docx]

**Supplementary Table S3. Detailed Patient Preferences for Interventions to Improve Comfort with Skin Grafting (N=475)**

| **Requested Intervention** | **Number of Patients Requesting (N)** | **Percentage (%)** | **Associated Post-op Anxiety Reduction (Subgroup Analysis)** |
| --- | --- | --- | --- |
| Questions & answers with attending burn physician | 464 | 97.7% | -- |
| Questions & answers with anesthesiologist | 253 | 53.3% | -- |
| **Combined Physician & Anesthesiologist Consultation** | **248** | **52.2%** | **32% lower post-op anxiety vs. single consultation (p=0.004)** |
| Questions & answers with nursing staff | 202 | 42.5% | -- |
| Access to statistical outcome data/surveys | 191 | 40.2% | -- |
| Literature about the surgical procedure | 179 | 37.7% | -- |
| Literature about the efficacy of skin grafting | 58 | 12.2% | -- |
| Other (e.g., peer support, virtual tour) | 35 | 7.4% | -- |

Note: Patients could select more than one intervention. The subgroup analysis for anxiety reduction compared patients who received consultations with both the attending physician and the anesthesiologist (n=248) to those who received a consultation with only one of these providers or neither (n=227). Postoperative anxiety was measured using the NRS.
