# Supplementary material for: Burn patients’ perceptions of skin grafting in China: a single-center retrospective cohort study with paired pre-post assessment
Source: Front Public Health. 2026 Jan 23;14:1754982. doi: 10.3389/fpubh.2026.1754982 (PMC12875900; doi:10.3389/fpubh.2026.1754982)
Supplement: Supplementary file 7 [file Supplementary_file_2.docx]

**Supplementary File 2: Survey Instruments and Methods Details**

**1. Questionnaire Overview**

The standardized self-report questionnaire used in this study was designed for burn patients undergoing skin grafting, aiming to systematically assess their psychological state, cognitive perceptions, and subjective experiences before and after surgery. The questionnaire consists of four core modules, with all items presented in Chinese.

**2. Questionnaire Modules and Items**

**2.1 Module A: Eligibility Screening and Demographic Information**

- **Purpose:** To confirm participant eligibility and collect baseline information.
- **Example Items:**
  1. Are you scheduled to undergo skin grafting surgery within this month? (Yes/No)
  2. Age: ____ years
  3. Gender: (Male/Female/Other)
  4. Primary site of the current burn: (Head/Face/Neck/Upper limb/Lower limb/Trunk/Buttocks/Perineum/Multiple sites)
  5. Planned number of skin grafting procedures: ____

**2.2 Module B: Graft Perception Assessment**

- **Purpose:** To quantify patients' knowledge, expectations, and emotional responses regarding skin grafting. Includes 10 structured items with binary (Yes/No or True/False) responses, used to calculate a composite 'Perception Score' (range 0–10, higher scores indicate better understanding/more positive perceptions).
- **Example Items (Knowledge Domain):**
  1. Skin grafting requires taking skin from another part of your body (donor site). (Yes/No)
  2. The grafted skin may feel temporarily numb after healing. (Yes/No)
  3. There is a risk of graft failure (approximately 5–8%). (Yes/No)
- **Example Items (Expectation Domain):**
  4. You expect the grafted skin to perfectly match the color of the surrounding skin. (Yes/No)
  5. You believe the grafted area will regain full function immediately after surgery. (Yes/No)
- **Example Items (Emotion Domain):**
  6. You feel hopeful about the outcome of this surgery. (Yes/No)
  7. You feel extreme fear about potential scarring or disfigurement. (Yes/No)

**2.3 Module C: Numerical Rating Scale (NRS) for Anxiety and Fear**

- **Purpose:** To assess the immediate intensity of anxiety and fear.
- **Scale Description:** Two separate 0–10 numerical rating scales.
  - **Anxiety NRS:** “Please use a number from 0 to 10 to describe your current level of anxiety about the surgery, where 0 means ‘no anxiety at all’ and 10 means ‘the most extreme anxiety’.” Score: ____
  - **Fear NRS:** “Please use a number from 0 to 10 to describe your current level of fear about the surgery, where 0 means ‘no fear at all’ and 10 means ‘the most extreme fear’.” Score: ____

**2.4 Module D: Hospital Anxiety and Depression Scale (HADS)**

- **Purpose:** To screen for clinically significant anxiety and depression symptoms.
- **Scale Description:** The Chinese version of the HADS was used, containing 14 items (7 for the anxiety subscale, 7 for the depression subscale). Each item is rated on a 4-point scale (0–3).
  - **Example Item (Anxiety):** “I feel tense or ‘wound up’” (Options: Most of the time / A lot of the time / From time to time / Not at all).
  - **Scoring:** Each subscale score ranges from 0 to 21. **A score ≥ 8** suggests probable clinically significant anxiety or depression.

**2.5 Module E: Postoperative Experience and Support Assessment**

- **Purpose:** To evaluate postoperative side effects, comfort-improving factors, and social support.
- **Example Items (Side Effects):**
  - “On the first day after surgery, did you experience any of the following?” (Multiple selection: Drowsiness / Surgical site pain / Nausea or vomiting / Transient confusion / Other ____)
- **Example Items (Comfort-Improving Factors):**
  - “Which of the following interventions do you believe would improve your comfort and acceptance of skin grafting?” (Multiple selection: In-depth discussion with the attending burn surgeon / Preoperative consultation with the anesthesiologist / Watching educational videos / Receiving printed educational materials with diagrams / Talking with peers who have undergone grafting / Emotional support from family)
- **Example Items (Social Support):**
  - “From whom did you primarily receive information or emotional support about grafting before or after surgery?” (Multiple selection: Family / Friends / Medical staff / Fellow patients / Media / None)

**3. Administration and Data Collection Procedure**

1. **Timing:** The questionnaire was administered by ward nurses at two fixed time points: **Preoperatively (T1, 1–3 days before surgery)** and **Postoperatively (T2, on the day of discharge)**.
2. **Mode:** Paper-based, self-administered. For patients with reading difficulties, trained nurses read the items aloud in a neutral, non-leading manner.
3. **Data Entry:** Completed forms were scanned and stored in the patients' electronic health records. The research team retrospectively extracted these data from the hospital database.
4. **Quality Control:** The database recorded the completion time for each questionnaire. Responses completed in **<120 seconds** were flagged as “inattentive responding” and excluded during data analysis to ensure quality.

**4. Scale References and Psychometric Properties**

- **Numerical Rating Scale (NRS):** Widely used for pain and affective symptom assessment, with good face validity and test-retest reliability. Validated for use in Chinese surgical populations.
- **Hospital Anxiety and Depression Scale (HADS):** Well-validated in Chinese populations, demonstrating good reliability and validity. In the present sample, internal consistency (Cronbach's α) was 0.78, indicating acceptable reliability.
